# Feasibility of Reidentifying Individuals in Large National Physical Activity Data Sets From Which Protected Health Information Has Been Removed With Use of Machine Learning

**DOI:** 10.1001/jamanetworkopen.2018.6040

**Published:** 2018-12-21

**Authors:** Liangyuan Na, Cong Yang, Chi-Cheng Lo, Fangyuan Zhao, Yoshimi Fukuoka, Anil Aswani

**Affiliations:** 1 Operations Research Center, Massachusetts Institute of Technology, Cambridge; 2 Department of Industrial Engineering and Operations Research, University of California, Berkeley; 3 Tsinghua-Berkeley Shenzhen Institute, Tsinghua University, Shenzhen, China; 4 Institute For Health & Aging, Department of Physiological Nursing, University of California, San Francisco

## Abstract

**Importance:**

Despite data aggregation and removal of protected health information, there is concern that deidentified physical activity (PA) data collected from wearable devices can be reidentified. Organizations collecting or distributing such data suggest that the aforementioned measures are sufficient to ensure privacy. However, no studies, to our knowledge, have been published that demonstrate the possibility or impossibility of reidentifying such activity data.

**Objective:**

To evaluate the feasibility of reidentifying accelerometer-measured PA data, which have had geographic and protected health information removed, using support vector machines (SVMs) and random forest methods from machine learning.

**Design, Setting, and Participants:**

In this cross-sectional study, the National Health and Nutrition Examination Survey (NHANES) 2003-2004 and 2005-2006 data sets were analyzed in 2018. The accelerometer-measured PA data were collected in a free-living setting for 7 continuous days. NHANES uses a multistage probability sampling design to select a sample that is representative of the civilian noninstitutionalized household (both adult and children) population of the United States.

**Exposures:**

The NHANES data sets contain objectively measured movement intensity as recorded by accelerometers worn during all walking for 1 week.

**Main Outcomes and Measures:**

The primary outcome was the ability of the random forest and linear SVM algorithms to match demographic and 20-minute aggregated PA data to individual-specific record numbers, and the percentage of correct matches by each machine learning algorithm was the measure.

**Results:**

A total of 4720 adults (mean [SD] age, 40.0 [20.6] years) and 2427 children (mean [SD] age, 12.3 [3.4] years) in NHANES 2003-2004 and 4765 adults (mean [SD] age, 45.2 [19.9] years) and 2539 children (mean [SD] age, 12.1 [3.4] years) in NHANES 2005-2006 were included in the study. The random forest algorithm successfully reidentified the demographic and 20-minute aggregated PA data of 4478 adults (94.9%) and 2120 children (87.4%) in NHANES 2003-2004 and 4470 adults (93.8%) and 2172 children (85.5%) in NHANES 2005-2006 (*P* < .001 for all). The linear SVM algorithm successfully reidentified the demographic and 20-minute aggregated PA data of 4043 adults (85.6%) and 1695 children (69.8%) in NHANES 2003-2004 and 4041 adults (84.8%) and 1705 children (67.2%) in NHANES 2005-2006 (*P* < .001 for all).

**Conclusions and Relevance:**

This study suggests that current practices for deidentification of accelerometer-measured PA data might be insufficient to ensure privacy. This finding has important policy implications because it appears to show the need for deidentification that aggregates the PA data of multiple individuals to ensure privacy for single individuals.

## Key Points

**Question:**

Is it possible to reidentify physical activity data that have had protected health information removed by using machine learning?

**Findings:**

This cross-sectional study used national physical activity data from 14 451 individuals from the National Health and Nutrition Examination Surveys 2003-2004 and 2005-2006. Linear support vector machine and random forests reidentified the 20-minute-level physical activity data of approximately 80% of children and 95% of adults.

**Meaning:**

The findings of this study suggest that current practices for deidentifying physical activity data are insufficient for privacy and that deidentification should aggregate the physical activity data of many people to ensure individuals’ privacy.

## Teaser

This cross-sectional study of National Health and Nutrition Examination Survey data sets evaluates the feasibility of reidentifying accelerometer-measured physical activity data that have had protected health information removed, using support vector machines and random forest methods from machine learning.

## Introduction

Policymakers^1,2^ have raised the possibility of identifying individuals or their actions based on activity data, whereas device manufacturers and exercise-focused social networks maintain that sharing deidentified data poses no privacy risks.^3,4,5^ Wearable device users are concerned with privacy issues,^6^ and ethical consequences have been discussed.^7,8^ There are also potentially legal requirements from the Health Insurance Portability and Accountability Act (HIPAA) on the privacy of activity data.^9,10,11^ One key unresolved question is whether it is possible to reidentify activity data. A better understanding on the feasibility of such reidentification will provide guidance to researchers, health care providers (ie, hospitals and physicians), and policymakers on creating practical privacy regulations for activity data.

Reidentification of data is not just theoretical but has been demonstrated in several contexts. For instance, demographics in an anonymized data set can function as a quasi-identifier that is capable of being used to reidentify individuals.^12^ Reidentification is also possible using online search data,^13^ movie rating data,^14^ social network data,^15^ and genetic data.^16^ However, a key feature in these examples is a type of data sparsity, specifically, a large number of characteristics for each individual, which leads to a diversity of combinations in such a way that any particular combination of the data is identifying. For example, individuals’ movie ratings are highly revealing because of the many permutations of likes and dislikes.^14^ As another example, the particular genetic sequence combinations (and especially single-nucleotide polymorphisms) of a single individual are unique and capable of identifying that individual.^16^

In contrast, physical activity data do not feature the type of data sparsity found in the above examples^12,13,14,15,16^ because health data from a single individual often exhibit high variability. For example, for heart rate, variability is a constant and expected feature in healthy and unhealthy individuals. However, this variability does not protect against reidentification. A previous study^17^ found that high temporal resolution data from wearable devices transform this variability into repeated patterns that can be used for reidentification. In response, commercial organizations have argued that aggregated sets of wearable device data (without the high resolution) cannot be reidentified.^3,4,5^ It was recently reported that location information from activity trackers could be used to identify the location of military sites.^18^ Although this is not strictly an example of reidentifying specific individuals, it is nonetheless an example of the potential loss of privacy attributable to sharing of physical activity data. As a result, many location data are no longer being shared by commercial organizations; however, to our knowledge, reidentification excluding location data has not been studied or demonstrated.

The primary aim of this study was to examine the feasibility of reidentifying activity data (collected from wearable devices) that have been partially aggregated. In this article, we specifically considered aggregations of an individual's activity data into walking intensity at the resolution of 20-minute intervals. This intensity represents a substantial level of aggregation compared with the raw digital accelerometer data that were used for reidentification in a previous study.^17^ We further studied other different levels of aggregation (from 15-minute intervals to 24-hour intervals) in the same manner.

**Figure 1.**
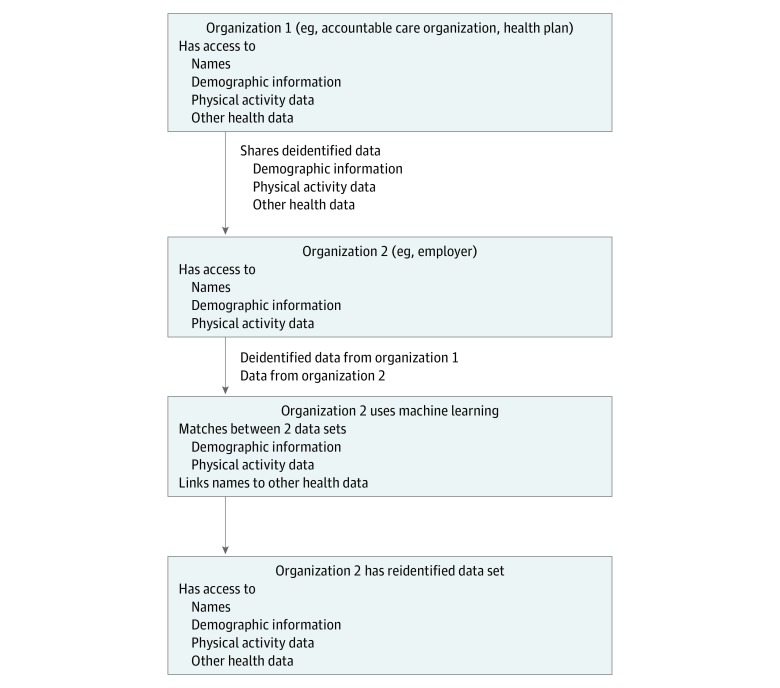
Threat Model for Reidentification of Health Data Using Physical Activity and Demographic Data

The scenario that we envisioned is summarized in Figure 1, and we gave one specific scenario to better describe the threat model considered in this article. This scenario involves an accountable care organization (ACO), such as the Kaiser Permanente network, that has stored their patients’ demographic data, complete health records, and physical activity data, which were collected as part of a weight loss intervention conducted by the ACO. This intervention involved recording physical activity data using a smartphone, activity tracker, or smartwatch. This scenario also involved an employer who has access to the names, demographic information, and physical activity data of their employees. The employer has access to physical activity data because they were collected by a smartphone, activity tracker, or smartwatch during the employees’ participation in a wellness program in exchange for a discount on health insurance premiums. There is a potential danger to privacy when the ACO shares deidentified data with the employer if the employer is able to reidentify the data using demographics and physical activity data. We evaluated the feasibility of this scenario by attempting to match a second data set of physical activity data and demographic information to a first data set of record numbers, physical activity data, and demographic information. From the standpoint of machine learning, matching record numbers is algorithmically and mathematically equivalent to matching names or other identifying information.

## Methods

### Design, Setting, and Participants

We conducted a cross-sectional study in 2018 (during which time data were being analyzed) using physical activity data measured by accelerometers (ActiGraph AM-7164) from the National Health and Nutrition Examination Survey (NHANES) from 2003-2004 and 2005-2006.^19,20^ NHANES is a yearly program conducted by the National Center for Health Statistics (NCHS) to track health and nutrition in the United States, and NCHS uses a multistage probability sampling design to select a participant sample that is representative of the diverse adult and child population in the United States. The NCHS Research Ethics Review Board approved all survey protocols for NHANES, and participants provided written informed consent before participating in NHANES. The Committee for Protection of Human Subjects at the University of California, Berkeley waived review of this study because it made use of only publicly available data. The reporting of this study conforms to the Strengthening the Reporting of Observational Studies in Epidemiology (STROBE) reporting guideline.^21^

### Physical Activity Data

One part of the NHANES data are the physical activity monitor (PAM) data, which were collected by accelerometers that objectively measure the intensity and duration of common locomotion activities, such as walking and jogging. The accelerometer was placed on an elasticized fabric belt, which was custom fitted for each study participant and worn on the right hip.^22^ Participants were instructed to wear the monitor every day for 7 days during all waking hours and to remove before swimming or bathing. The PAM data were obtained from a subset of individuals older than 6 years. The PAM data for 7176 individuals in the NHANES 2003-2004 survey^19^ and 7455 individuals in the 2005-2006^20^ survey are available. Although these PAM data have been used to evaluate whether the US population follows guidelines on recommended levels of physical activity,^23,24^ our study is the first, to our knowledge, to use the PAM data to study privacy questions.

The raw PAM data for NHANES 2003-2004 and 2005-2006 consist of 1-minute resolution intensity data measured during 7 days for 9601 adults and 5030 children. Although data collection did not occur in the same calendar week for all participants, the accelerometers were programmed to start collecting data at exactly 12:01 am on the first day of the 7 consecutive days for each participant. The time of each measurement was recorded using the local time of the participant. Because of large differences between weekday and weekend activity patterns, we used Monday through Friday data only for our study. We removed the data of participants with incomplete activity data (ie, the recorded data did not span the full 7 days) or with outlying activity data. We defined outlying activity data to be those that have zero variance among intensity values because it is incorrect to have constant recorded intensity value throughout the entire week.

### Demographic Data

Demographic data were collected for all NHANES participants, who were interviewed about themselves and their household members. Demographic information was collected using a computer-assisted personal interviewing method at participants’ home before their health examination. The participants self-classified their race/ethnicity from a set of options defined by NCHS. Race/ethnicity was assessed to allow examination of racial/ethnic differences in disease prevalence.

In this study, we used 6 demographic variables: age at screening adjudicated, sex, educational level, annual household income, race/ethnicity, and country of birth. We analyzed adults’ and children’s data separately. We used the current National Institutes of Health’s definition of children as individuals younger than 18 years.^25^ NHANES defined children as individuals younger than 19 years, and thus we appropriately recategorized the educational level for individuals 18 and 19 years of age. For educational level and annual household income, we imputed missing entries with the mean of the corresponding valid values among the PAM participants in the data sets rounded to the nearest integer.

### Partial Data Aggregation Process

The raw PAM data consist of record numbers (with 1 record number assigned to each individual) that are associated with the demographic data described above and with 1-minute resolution activity data (consisting of walking intensity) for 7 consecutive days. For this study, we only used the data from Monday through Friday because activity patterns on the weekend substantially differ from patterns on the weekday. To partially aggregate these data, we reduced the time resolution of the physical activity data to 20-minute intervals by summing the 1-minute resolution activity data for each 20-minute interval. Furthermore, we constructed 2 partially aggregated data sets from the raw PAM data. The first was the training data, which consisted of the record numbers, demographic data, and physical activity data aggregated into 20-minute intervals for Monday through Wednesday. The second was the testing data, which consisted of the record numbers, demographic data, and physical activity data aggregated into 20-minute intervals for Thursday and Friday. The difference between the training and testing data is that they have physical activity data from different days.

### Reidentification Procedure

**Figure 2.**
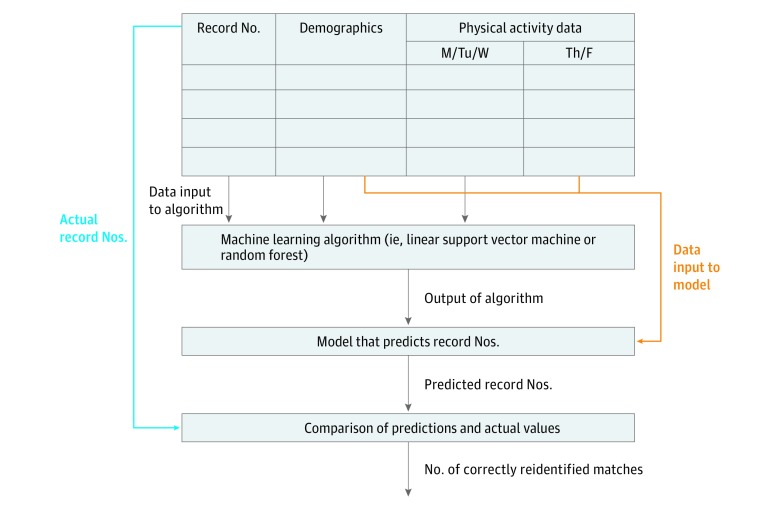
Block Diagram Showing the Main Steps of the Reidentification Procedure

To evaluate the feasibility of reidentifying physical activity data, we initially input the entire training data into machine learning algorithms to build models in which record number was the response variable and physical activity data and demographics were the predictor variables. Next, we input the demographic and physical activity data (but not the record numbers) from the testing data into the models to make predictions of record numbers. We evaluated the accuracy of the models by counting how many of the predicted record numbers matched the actual record numbers in the testing data. This was a test of reidentification because record numbers are considered to be protected health information in certain settings.^11^ A block diagram with the steps of this procedure is shown in Figure 2.

Our procedure to construct the model was to build a separate multiclass classifier^26,27,28,29^ for each combination of demographic attributes. The response variable in the multiclass classifier was the record number, and the predictors were the partially aggregated physical activity and demographic data from the training data. To predict a record number given the partially aggregated physical activity of a single record number from the testing data, we used the multiclass classifier corresponding to the demographics of these particular physical activity data to make a prediction of the record number. We used 2 different machine learning algorithms for multiclass classification that we tested when building the models.

#### Linear Support Vector Machine 

The linear support vector machine (SVM)^26^ is a popular classification algorithm that has fast computation speed, is easily interpretable, and has good accuracy. We used the LiblineaR package in R (R Foundation for Statistical Computing) to build linear SVMs for multiclassification using the training data, and this package uses the one–vs–the rest algorithm^27^ to match multiple classes. Cross-validation was used to choose among model types with different combinations of loss functions and regularization parameters. The 5 possible model types were L2-regularized, L2-loss support vector classification (dual); L2-regularized, L2-loss support vector classification (primal); L2-regularized, L1-loss support vector classification; support vector classification by Crammer and Singer^30^; and L1-regularized, L2-loss support vector classification.

#### Random Forests

Random forests^28^ are an ensemble learning method that combines a large number of decision trees (and implicitly uses the one-vs-one algorithm^29^) to make predictions. Although random forest models are difficult to interpret, this approach is one of the most successful machine learning techniques because it often has the highest accuracy of any machine learning method across many diverse data sets. We used the randomForest package in R to build a random forest model. Cross-validation was used to tune the number of variables randomly sampled as candidates.

### Statistical Analysis

We evaluated the accuracy of each model by counting how many of the predicted record numbers matched the actual record numbers in the testing data, and we conducted a hypothesis test under the null hypothesis that the number of matches follow a binomial distribution with success probability corresponding to the inverse of the number of individuals. This is a test of the null hypothesis that all matches were attributable to pure chance, and it is a test of reidentification because we were quantifying the ability for various models computed by machine learning algorithms to match activity data to unique identifiers.^11^ Bonferroni correction was used to adjust for multiple testing, and we report the Bonferroni-adjusted 2-sided *P* values in the Results section. We used a significance level of *P* < .05 when interpreting our statistical results.

### Sensitivity Analysis

Several other scenarios were possible in terms of what information was available for the training and testing data. Therefore, we conducted a sensitivity analysis to understand the feasibility of reidentifying physical activity data in these alternative scenarios. First, we conducted separate analysis for partial aggregation of the PAM data into different intervals, ranging from 15-minute to 24-hour intervals to test how the coarseness of the partial aggregation affects accuracy of deidentification. Second, we conducted separate analysis using different selections of days of the week for the training and testing data. The original analysis used Monday through Wednesday for training data and Thursday and Friday for testing data. This second sensitivity analysis was a test of how accuracy changes, depending on days of the week that are used for training or testing. Third, we conducted another analysis in which there was only a partial overlap in the record numbers present in the training and testing data. In the original analysis, all record numbers in the training data were present in the testing data. In this sensitivity analysis, 60% of the record numbers in the entire data set were present in both the training and the testing data, 20% of the record numbers were present only in the training data, and 20% of the record numbers were present only in the testing data. The model predictions were calibrated to predict no match with any record number in the testing data when the confidence in a particular prediction was low, and a correct identification of no match on the testing data was counted as a correct reidentification match.

## Results

### Demographics

Our exclusion criteria resulted in the removal of the data on 20 children and 129 adults with incomplete activity data for NHANES 2003-2004 and 9 children and 22 adults for unusual outliers for NHANES 2005-2006. The data removed from our analysis represent 180 (1.2%) of the total 14 631 individuals in the full physical activity data sets. In this analysis, 4720 adults (mean [SD] age, 40.0 [20.6] years) and 2427 children (mean [SD] age, 12.3 [3.4] years) in NHANES 2003-2004 and 4765 adults (mean [SD] age, 45.2 [19.9] years) and 2539 children (mean [SD] age, 12.1 [3.4] years) in NHANES 2005-2006 were included. Table 1 gives the sociodemographic characteristics of the study participants.

**Table 1.** Sociodemographic Characteristics of the Physical Activity Monitor Data Subset^a^

Characteristic	NHANES 2003-2004		NHANES 2005-2006	
	Adults (n = 4720)	Children (n = 2427)	Adults (n = 4765)	Children (n = 2539)
Age, mean (SD), y	40.0 (20.6)	12.3 (3.4)	45.2 (19.9)	12.1 (3.4)
Sex				
Male	2274 (48.2)	1236 (50.9)	2272 (47.7)	1264 (49.8)
Female	2446 (51.8)	1191 (49.1)	2493 (52.3)	1275 (50.2)
Educational level				
High school or less	2645 (56.0)	2425 (99.9)	2568 (53.9)	2538 (99.9)
More than high school	2069 (43.8)	2 (0.1)	2192 (46.0)	1 (0.1)
Missing	6 (0.1)	0	5 (0.1)	0
Annual household income, $				
<25 000	1574 (33.3)	767 (31.6)	1368 (28.7)	698 (27.5)
25 000-55 000	1831 (38.8)	935 (38.4)	1793 (37.6)	912 (35.9)
>55 000	1315 (27.9)	725 (30.0)	1604 (33.7)	929 (36.6)
Race/ethnicity				
Hispanic	1160 (24.6)	839 (34.6)	1150 (24.1)	907 (35.7)
White	2392 (50.7)	636 (26.2)	2267 (47.6)	667 (26.3)
Black	973 (20.6)	856 (35.3)	1156 (24.3)	815 (32.1)
Other	195 (4.1)	96 (3.9)	192 (4.0)	150 (5.9)
Country of birth				
United States	3774 (79.9)	2200 (90.6)	3747 (78.6)	2264 (89.2)
Outside the United States	945 (20.0)	227 (9.4)	1016 (21.3)	275 (10.8)
Missing	1 (0.1)	0	2 (0.1)	0
Daily physical activity intensity, counts, mean (SD)^b^				
Monday	172.0 (762.1)	285.1 (951.4)	208.6 (1237.0)	277.4 (1021.3)
Tuesday	183.3 (954.8)	279.4 (1072.2)	225.0 (1425.7)	283.6 (1214.8)
Wednesday	177.9 (934.1)	269.4 (1108.6)	230.9 (1536.8)	296.1 (1478.4)
Thursday	176.0 (1009.7)	263.4 (1146.0)	229.9 (1572.0)	334.3 (1883.8)
Friday	180.4 (1100.9)	257.1 (1186.3)	245.6 (1789.1)	328.0 (1949.2)

Abbreviation: NHANES, National Health and Nutrition Examination Survey.

^a^ Data are present as number (percentage) of participants unless otherwise indicated.

^b^ The ActiGraph AM-7164 returns data in digital units of counts.

### Reidentification

Table 2 gives the number of correct reidentification matches using physical activity data at 20-minute intervals. The random forest algorithm successfully reidentified the demographic and 20-minute aggregated physical activity data of 4478 adults (94.9%) and 2120 children (87.4%) in NHANES 2003-2004 and 4470 adults (93.8%) and 2172 children (85.5%) in NHANES 2005-2006 (*P* < .001 for all). The linear SVM algorithm successfully reidentified the demographic and 20-minute aggregated PA data of 4043 adults (85.6%) and 1695 children (69.8%) in NHANES 2003-2004 and 4041 adults (84.8%) and 1705 children (67.2%) in NHANES 2005-2006 (*P* < .001 for all).

**Table 2.** Number of Correctly Reidentified Matches in Testing Data With Physical Activity Data Partially Aggregated Into 20-Minute Intervals

Machine Learning Algorithm	No. (%) of Adults^a^		No. (%) of Children^a^	
	Demographics Only	Demographics With Physical Intensity	Demographics Only	Demographics With Physical Intensity
**NHANES 2003-2004**				
Linear SVM	3880 (81.2)	4043 (85.6)	1496 (61.6)	1695 (69.8)
Random Forest		4478 (94.9)		2120 (87.4)
**NHANES 2005-2006**				
Linear SVM	3827 (80.3)	4041 (84.8)	1514 (59.6)	1705 (67.2)
Random Forest		4470 (93.8)		2172 (85.5)

Abbreviations: NHANES, National Health and Nutrition Examination Survey; SVM, support vector machine.

^a^ *P* < .001.

Table 3 gives the results of the sensitivity analysis of using different partial aggregation intervals, and these matching accuracies are plotted in eFigures 1 through 4 in the Supplement. Although matching accuracy decreased with longer intervals, the matches were statistically significant because of *P* < .001 for the different intervals, machine learning algorithms, and data sets. For instance, the random forest algorithm successfully reidentified the demographic and 15-minute aggregated physical activity data of 4451 adults (94.3%) and 2116 (87.2%) of children in NHANES 2003-2004. The random forest algorithm successfully reidentified the demographic and 24-hour aggregated physical activity data of 4106 adults (87.0%) and 1704 (70.2%) of children in NHANES 2003-2004. The linear SVM algorithm successfully reidentified the demographic and 15-minute aggregated physical activity data of 4026 adults (84.5%) and 1706 (67.2%) of children in NHANES 2005-2006. The linear SVM algorithm successfully reidentified the demographic and 24-hour aggregated physical activity data of 3960 adults (83.1%) and 1584 (62.4%) of children in NHANES 2005-2006.

**Table 3.** Percentage of Correctly Reidentified Matches at Different Time Resolutions of Partial Aggregation of Physical Activity Data

Machine Learning Algorithm	Correctly Reidentified Matches, %									
	15 min	20 min	30 min	1 h	2 h	4 h	6 h	8 h	12 h	24 h
**Adults in NHANES 2003-2004**										
Demographics only^a^	81.1^b^									
Physical activity										
Linear SVM^c^	0	0	0	0.02	0	0.04	0	0.06	0.02	0.02
Random forest^a^	5.97	5.93	6.52	5.61	4.24	2.14	1.14	0.74	0.23	0.23
Demographics and physical activity										
Linear SVM^a^	85.2	85.6	87.0	86.9	87.7	87.4	86.8	86.7	85.7	84.8
Random forest^a^	94.3	94.9	94.5	94.0	93.0	91.7	91.3	90.6	89.0	87.0
**Adults in NHANES 2005-2006**										
Demographics only^a^	80.3^b^									
Physical activity										
Linear SVM^c^	0	0.02	0.02	0	0.02	0	0.02	0.02	0.02	0.02
Random forest	6.40^a^	6.48^a^	6.55^a^	6.04^a^	4.55^a^	2.29^a^	1.09^a^	0.55^a^	0.23^a^	0.08^b^
Demographics and physical activity										
Linear SVM^a^	84.5	84.8	84.7	85.1	86.3	86.1	85.9	85.5	85.0	83.1
Random forest^a^	93.5	93.8	93.2	92.8	91.9	91.0	90.5	89.4	87.9	85.8
**Children in NHANES 2003-2004**										
Demographics only^a^	61.6^b^									
Physical activity										
Linear SVM^c^	0.08	0	0.04	0.04	0	0.04	0	0.08	0.04	0.04
Random Forest	11.1^a^	10.5^a^	11.0^a^	7.83^a^	4.45^a^	1.98^a^	0.95^a^	0.58^a^	0.37^a^	0.04^b^
Demographics and physical activity										
Linear SVM^a^	70.3	69.8	70.8	71.5	68.9	69.9	67.0	68.6	67.5	64.3
Random forest^a^	87.2	87.4	87.1	84.8	83.1	80.0	78.6	76.9	73.8	70.2
**Children in NHANES 2005-2006**										
Demographics only^a^	59.4^b^									
Physical activity										
Linear SVM^c^	0.08	0	0.04	0.08	0	0.08	0.04	0	0.08	0
Random forest^a^	10.6	11.5	10.0	7.17	4.25	1.81	1.02	0.91	0.32	0.32
Demographics and physical activity										
Linear SVM^a^	67.2	67.2	67.0	67.2	66.0	67.0	66.4	67.0	64.7	62.4
Random forest^a^	84.8	85.5	84.4	82.6	80.3	78.2	75.9	74.5	70.9	67.3

Abbreviations: NHANES, National Health and Nutrition Examination Survey; SVM, support vector machine.

^a^ *P* < .001.

^b^ The demographics-only results are not subject to any time; thus, the same numbers are independent of the time columns

^c^ *P* > .99.

Both random forest and linear SVM using demographics and physical activity improved the matching accuracy from the classifier using only demographics, which was confirmed by the hypothesis testing. Accuracy when using only physical activity data was low. With the use of demographic data alone, we were able to successfully reidentify 3880 adults (81.2%) and 1496 children (61.6%) in NHANES 2003-2004 and 3827 adults (80.3%) and 1514 children (59.6%) in NHANES 2005-2006.

As indicated in eTable 1 in the Supplement, changing the days of the week used for the training and testing data did not change the matching accuracy. For instance, the random forest algorithm was able to successfully reidentify 4478 (94.9%), 4383 (92.8%), 4413 (93.5%), 4405 (93.3%), 4400 (93.2%), and 4402 (93.2%) of adults in NHANES 2003-2004 when using Thursday and Friday, Monday and Thursday, Tuesday and Thursday, Tuesday and Friday, Wednesday and Thursday, and Wednesday and Friday, respectively, for the testing data.

Results in eTable 2 in the Supplement show that limiting the demographics to only age and sex substantially decreased matching accuracy. For instance, the random forest algorithm using age, gender, and physical activity data was able to successfully reidentify only 1511 adults (32.0%) in NHANES 2003-2004. The results in eTable 3 in the Supplement show that introducing an artificial nonoverlap in the record numbers in the training and testing data did not substantially change the matching accuracy. For instance, the random forest algorithm was able to successfully reidentify 3240 adults (90.5%) in the testing data constructed from NHANES 2003-2004. Recall that the denominator is smaller for this third sensitivity analysis because only 80% of all adults were included in the testing data.

## Discussion

Policymakers^1,2^ have been concerned about the possibility of identifying individuals or their actions based on activity data, whereas device manufacturers and exercise-focused social networks believe that sharing deidentified physical activity data poses no privacy risks.^3,4,5^ Because it was recently reported that location information from activity trackers could be used to identify the location of military sites,^18^ these groups have begun to restrict which location data are shared. However, device manufacturers continue to share deidentified physical activity data with individuals’ employers, advertisers, and health care organizations.^10^ Thus, it is vital to be able to quantify the privacy risks from sharing such data.

Our results suggest that partially aggregated PAM data with geographic and protected health information removed can be reidentified using machine learning. The random forest algorithm successfully reidentified the demographic and 20-minute aggregated physical activity data of 4478 adults (94.9%) and 2120 children (87.4%) in NHANES 2003-2004 and 4470 adults (93.8%) and 2172 children (85.5%) in NHANES 2005-2006. Moreover, partial aggregation of the physical activity data only slightly decreased the accuracy of reidentification. When the activity data were aggregated into daily-level data, we were still able to reidentify a large fraction of subjects. For instance, the random forest algorithm successfully reidentified the demographic and 24-hour aggregated physical activity data of 4106 adults (87.0%) and 1704 children (70.2%) in NHANES 2003-2004, and the linear SVM algorithm successfully reidentified the demographic and 24-hour aggregated physical activity data of 3960 adults (83.1%) and 1584 (62.4%) of children in NHANES 2005-2006. As a baseline, using only demographic information allowed us to reidentify 3880 adults (81.2%) and 1496 children (61.6%) in NHANES 2003-2004 and 3827 adults (80.3%) and 1514 children (59.6%) in NHANES 2005-2006. This finding demonstrates that physical activity data may provide additional identifying information about each individual. Furthermore, our reidentification process does not make use of any data disallowed for sharing by the Safe Harbor determination method of the HIPAA) privacy rule.^11^

These findings show that sharing deidentified physical activity data may constitute a serious privacy risk, which is problematic because employers, advertisers, and other groups may receive deidentified physical activity data.^10^ From a policy perspective, the number of individuals in the NHANES PAM data that we used are representative of the number of employees in medium-sized businesses. A recent legal analysis^10^ analyzed possible privacy concerns arising from such employers and their access to physical activity data, and our analysis indicated that such data sharing with employers is a particularly serious issue because of our demonstration of the ability to deidentify physical activity data.

We believe that our findings have important policy implications. First, policymakers should consider developing regulations to restrict the sharing of activity data by device manufacturers. Although these organizations are collecting and sharing sensitive health data, they are likely not bound by existing regulations in most circumstances. Second, privacy risks from sharing activity data can be somewhat mitigated by aggregating data not only in time but also across individuals of largely different demographics. This consideration is particularly important for governmental organizations making public releases of large national health data sets, such as NHANES.

### Strengths and Limitations

The strengths of our study are that we demonstrated the ability to reidentify physical activity data using a large PAM data set that is representative of the US population and standard machine learning algorithms readily available in common software packages. Our study was also the first, to our knowledge, to examine privacy questions using the publicly available data sets. In addition, we evaluated how aggregation of physical activity data in time affects the ability to reidentify the data.

There are a number of limitations to our study. One limitation is that we required the use of demographic information to achieve high accuracy. Thus, the findings of our study may not be generalizable when demographic information is not shared with the PAM data. Matching accuracy using only age and sex demographics also reduced accuracy significantly. Another limitation is that time information for the PAM data was provided in local time; therefore, we were not able to study the effect of shifting all data to a common time (eg, Greenwich mean time). It is possible that the achieved high accuracy was attributable to matching circadian patterns and that if all data were provided at a common time, the accuracy may have been lower because of breaking the link to circadian rhythms. An additional limitation concerns the amount of nonoverlap in the record numbers between the training and testing data. Our sensitivity analysis considered a nonoverlap in which 20% of the record numbers in the training data were not in the testing data and 20% of the record numbers in the testing data were not in the training data. The matching accuracy is likely to substantially decrease as the amount of nonoverlap increases.

## Conclusions

Using large national physical activity data sets, we found that machine learning successfully reidentified the physical activity data of most children and adults when using 20-minute data with several pieces of demographic information. Partial aggregation of the data over time (eg, reidentifying daily-level physical activity data) did not significantly reduce the accuracy of the reidentification. These results suggest that current practices for deidentification of PAM data might be insufficient to ensure privacy and that there is a need for deidentification that aggregates the physical activity data of multiple individuals to ensure privacy for single individuals.

## Notes

Supplement. **eFigure 1.** Percentage of Correctly Reidentified Matches at Different Time Resolutions of Partial Aggregation of Physical Activity Data for Adults in NHANES 2003-2004 **eFigure 2.** Percentage of Correctly Reidentified Matches at Different Time Resolutions of Partial Aggregation of Physical Activity Data for Adults in NHANES 2005-2006 **eFigure 3.** Percentage of Correctly Reidentified Matches at Different Time Resolutions of Partial Aggregation of Physical Activity Data for Children in NHANES 2003-2004 **eFigure 4.** Percentage of Correctly Reidentified Matches at Different Time Resolutions of Partial Aggregation of Physical Activity Data for Children in NHANES 2005-2006 **eTable 1.** Number of Correctly Reidentified Matches in Testing Data, with Physical Activity Data Partially Aggregated into 20 Minute Intervals, When Different Days of the Week Are Used for the Training and Testing Data **eTable 2.** Number of Correctly Reidentified Matches in Testing Data, with Physical Activity Data Partially Aggregated into 20 Minute Intervals, When the Only Demographic Data Used is Age and Gender **eTable 3.** Number of Correctly Reidentified Matches in Testing Data, with Physical Activity Data Partially Aggregated into 20 Minute Intervals, When There is an Artificial Non-Overlap in the Record Numbers in the Testing and Training Data
