# Supplementary material for: Feasibility of Reidentifying Individuals in Large National Physical Activity Data Sets From Which Protected Health Information Has Been Removed With Use of Machine Learning
Source: JAMA Netw Open. 2018 Dec 21;1(8):e186040. doi: 10.1001/jamanetworkopen.2018.6040 (PMC6324329; doi:10.1001/jamanetworkopen.2018.6040)
Supplement: Supplement. — eFigure 1. Percentage of Correctly Reidentified Matches at Different Time Resolutions of Partial Aggregation of Physical Activity Data for Adults in NHANES 2003-2004 eFigure 2. Percentage of Correctly Reidentified Matches at Different Time Resolutions of Partial Aggregation of Physical Activity Data for Adults in NHANES 2005-2006 eFigure 3. Percentage of Correctly Reidentified Matches at Different Time Resolutions of Partial Aggregation of Physical Activity Data for Children in NHANES 2003-2004 eFigure 4. Percentage of Correctly Reidentified Matches at Different Time Resolutions of Partial Aggregation of Physical Activity Data for Children in NHANES 2005-2006 eTable 1. Number of Correctly Reidentified Matches in Testing Data, with Physical Activity Data Partially Aggregated into 20 Minute Intervals, When Different Days of the Week Are Used for the Training and Testing Data eTable 2. Number of Correctly Reidentified Matches in Testing Data, with Physical Activity Data Partially Aggregated into 20 Minute Intervals, When the Only Demographic Data Used is Age and Gender eTable 3. Number of Correctly Reidentified Matches in Testing Data, with Physical Activity Data Partially Aggregated into 20 Minute Intervals, When There is an Artificial Non-Overlap in the Record Numbers in the Testing and Training Data [file jamanetwopen-1-e186040-s001.pdf]

Supplementary Online Content

Na L, Yang C, Lo C-C, Zhao F, Fukuoka Y, Aswani A. Feasibility of reidentifying individuals in large national physical activity data sets from which protected health information has been removed with use of machine learning. *JAMA Netw Open.* 2018;1(8):e186040. doi:10.1001/jamanetworkopen.2018.6040

eFigure 1. Percentage of Correctly Reidentified Matches at Different Time Resolutions of Partial Aggregation of Physical Activity Data for Adults in NHANES 2003-2004

eFigure 2. Percentage of Correctly Reidentified Matches at Different Time Resolutions of Partial Aggregation of Physical Activity Data for Adults in NHANES 2005-2006

eFigure 3. Percentage of Correctly Reidentified Matches at Different Time Resolutions of Partial Aggregation of Physical Activity Data for Children in NHANES 2003-2004

eFigure 4. Percentage of Correctly Reidentified Matches at Different Time Resolutions of Partial Aggregation of Physical Activity Data for Children in NHANES 2005-2006

eTable 1. Number of Correctly Reidentified Matches in Testing Data, with Physical Activity Data Partially Aggregated into 20 Minute Intervals, When Different Days of the Week Are Used for the Training and Testing Data

eTable 2. Number of Correctly Reidentified Matches in Testing Data, with Physical Activity Data Partially Aggregated into 20 Minute Intervals, When the Only Demographic Data Used is Age and Gender

eTable 3. Number of Correctly Reidentified Matches in Testing Data, with Physical Activity Data Partially Aggregated into 20 Minute Intervals, When There is an Artificial Non-Overlap in the Record Numbers in the Testing and Training Data

This supplementary material has been provided by the authors to give readers additional information about their work.

© 2018 Na L et al. *JAMA Network Open.*

eFigure 1. Percentage of Correctly Reidentified Matches at Different Time Resolutions of Partial Aggregation of Physical Activity Data for Adults in NHANES 2003-2004

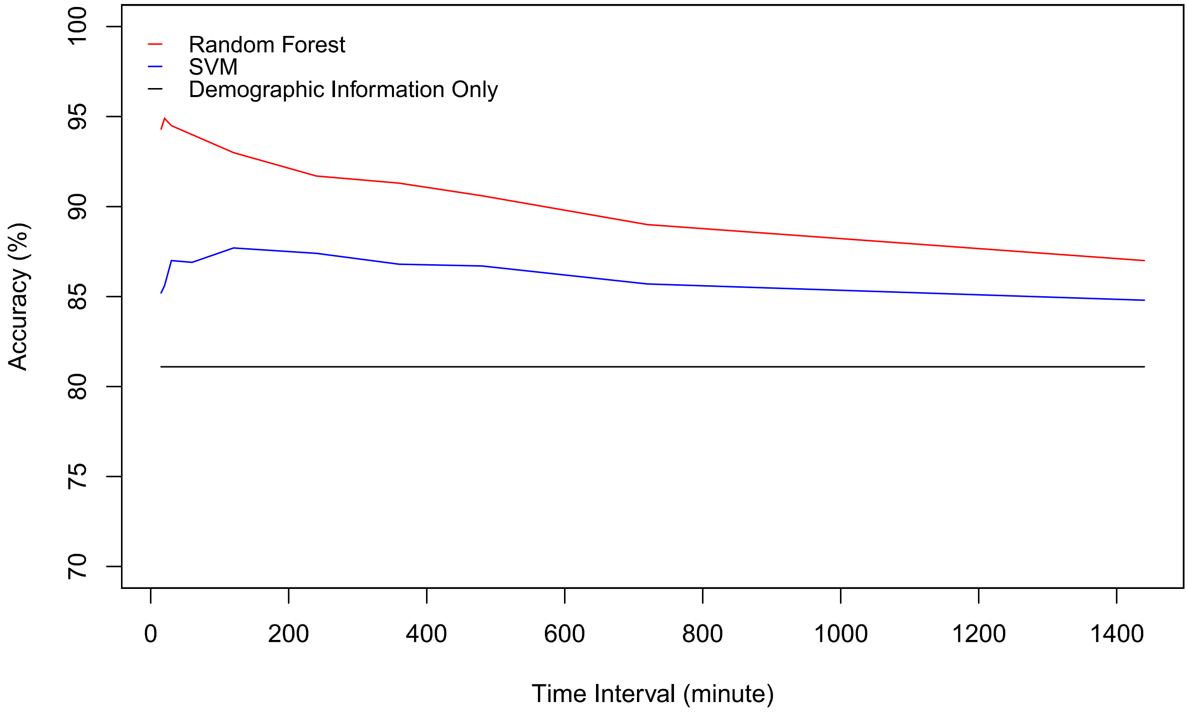

© 2018 Na L et al. *JAMA Network Open.*

eFigure 2. Percentage of Correctly Reidentified Matches at Different Time Resolutions of Partial Aggregation of Physical Activity Data for Adults in NHANES 2005-2006

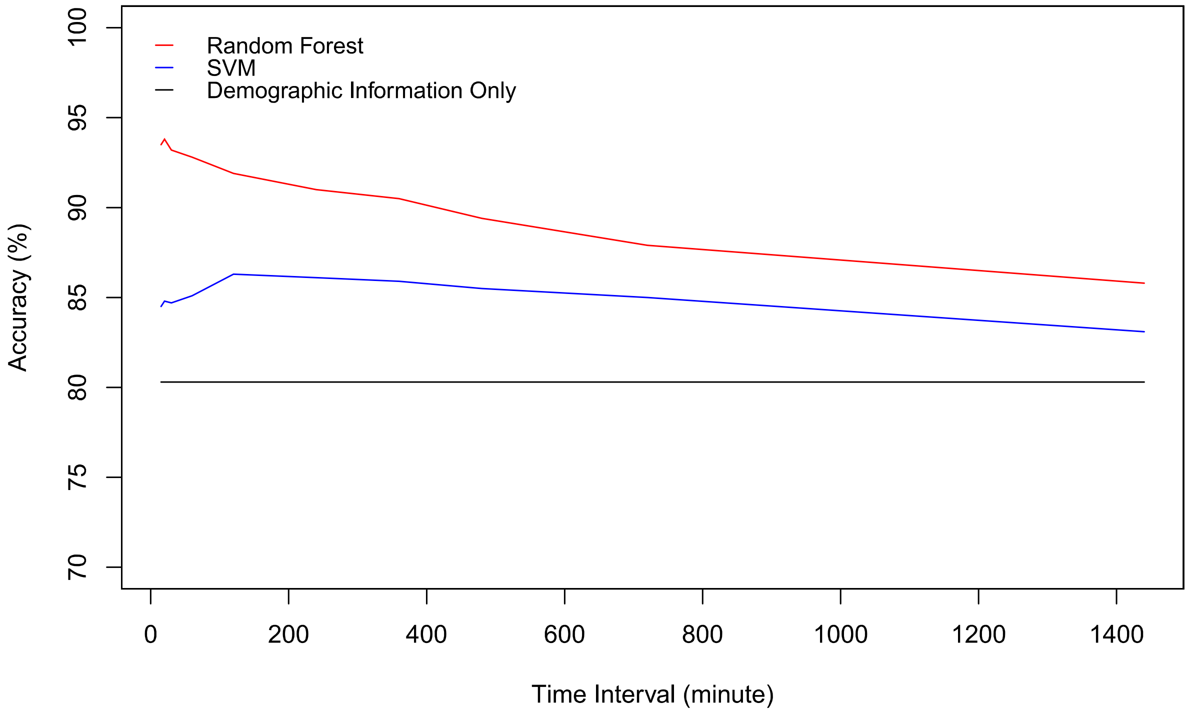

© 2018 Na L et al. *JAMA Network Open.*

eFigure 3. Percentage of Correctly Reidentified Matches at Different Time Resolutions of Partial Aggregation of Physical Activity Data for Children in NHANES 2003-2004

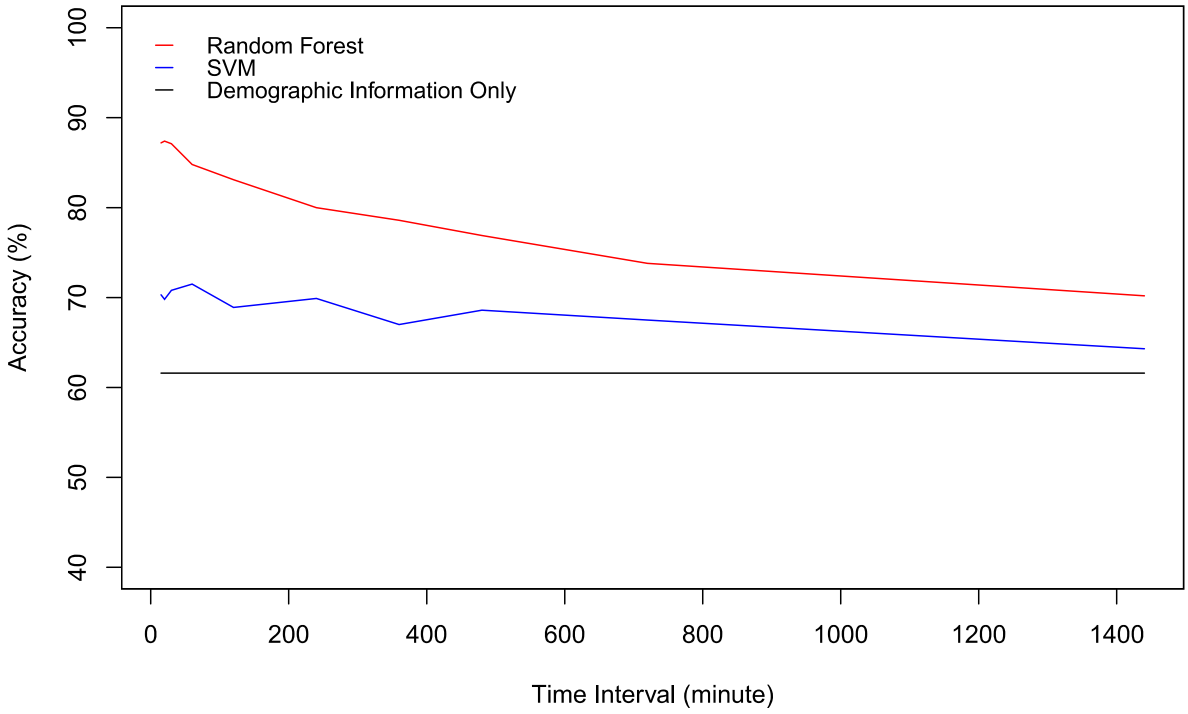

© 2018 Na L et al. *JAMA Network Open.*

eFigure 4. Percentage of Correctly Reidentified Matches at Different Time Resolutions of Partial Aggregation of Physical Activity Data for Children in NHANES 2005-2006

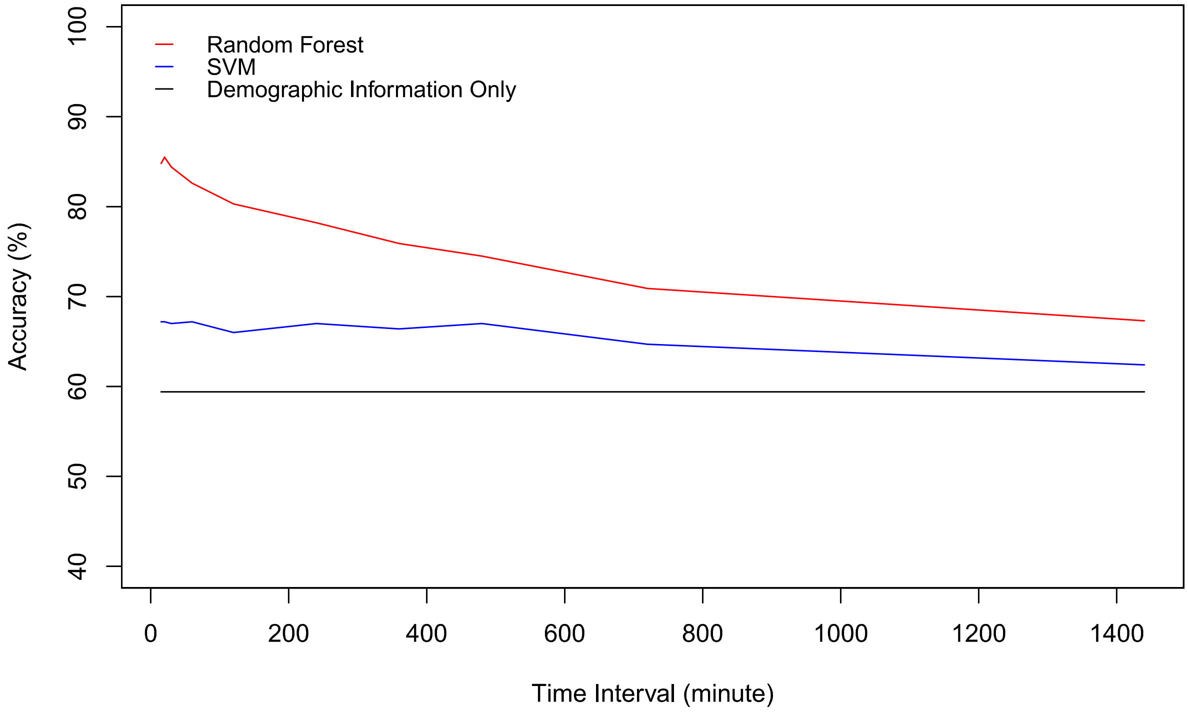

© 2018 Na L et al. *JAMA Network Open.*

eTable 1. Number of Correctly Reidentified Matches in Testing Data, with Physical Activity Data Partially Aggregated into 20 Minute Intervals, When Different Days of the Week Are Used for the Training and Testing Data

Sample/ Year	Machine Learning Algorithm	Days Used for Testing Data				
		Mon, Thu	Tue, Thu	Tue, Fri	Wed, Thu	Wed, Fri
		No. (%)	No. (%)	No. (%)	No. (%)	No. (%)
Adults in NHANES 2003-2004	Linear SVM	4036 (85.5)[a]	4049 (85.8)[a]	4063 (86.1)[a]	4033 (85.4)[a]	4034 (85.4)[a]
	Random Forest	4383 (92.8)[a]	4413 (93.5)[a]	4405 (93.3)[a]	4400 (93.2)[a]	4402 (93.2)[a]
Adults in NHANES 2005-2006	Linear SVM	4018 (84.3)[a]	4024 (84.4)[a]	4030 (84.6)[a]	4055 (85.1)[a]	4050 (85.0)[a]
	Random Forest	4370 (91.7)[a]	4403 (92.4)[a]	4409 (92.5)[a]	4392 (92.1)[a]	4384 (92.0)[a]
Children in NHANES 2003-2004	Linear SVM	1664 (68.6)[a]	1715 (70.7)[a]	1704 (70.2)[a]	1680 (69.2)[a]	1685 (69.4)[a]
	Random Forest	2019 (83.2)[a]	2099 (86.5)[a]	2068 (85.2)[a]	2084 (85.9)[a]	2065 (85.1)[a]
Children in NHANES 2005-2006	Linear SVM	1699 (66.9)[a]	1708 (67.2)[a]	1721 (67.8)[a]	1692 (66.6)[a]	1727 (68.0)[a]
	Random Forest	2089 (82.3)[a]	2145 (84.5)[a]	2114 (83.3)[a]	2111 (83.1)[a]	2120 (83.5)[a]

Abbreviations: NHANES, National Health and Nutrition Examination Survey; SVM = Support Vector Machine
[a] $P < 0.001$

© 2018 Na L et al. *JAMA Network Open.*

eTable 2. Number of Correctly Reidentified Matches in Testing Data, with Physical Activity Data Partially Aggregated into 20 Minute Intervals, When the Only Demographic Data Used is Age and Gender

**NHANES 2003-2004**				
**Machine Learning Algorithm**	**Adults: Age and Gender Only**	**Adults: Age and Gender with Physical Intensity**	**Children: Age and Gender Only**	**Children: Age and Gender with Physical Intensity**
	**No. (%)**	**No. (%)**	**No. (%)**	**No. (%)**
Linear SVM	136 (2.9)[a]	152 (3.2)[a]	24 (1.0)[a]	21 (0.9)[a]
Random Forest		1511 (32.0)[a]		619 (25.5)[a]
**NHANES 2005-2006**				
**Machine Learning Algorithm**	**Adults: Age and Gender Only**	**Adults: Age and Gender with Physical Intensity**	**Children: Age and Gender Only**	**Children: Age and Gender with Physical Intensity**
	**No. (%)**	**No. (%)**	**No. (%)**	**No. (%)**
Linear SVM	136 (2.9)[a]	168 (3.5)[a]	24 (0.9)[a]	26 (1.0)[a]
Random Forest		1597 (33.5)[a]		669 (26.3)[a]

Abbreviations: NHANES, National Health and Nutrition Examination Survey;SVM = Support Vector Machine
[a] $P < 0.001$

© 2018 Na L et al. *JAMA Network Open.*

eTable 3. Number of Correctly Reidentified Matches in Testing Data, with Physical Activity Data Partially Aggregated into 20 Minute Intervals, When There is an Artificial Non-Overlap in the Record Numbers in the Testing and Training Data

**NHANES 2003-2004**				
**Machine Learning Algorithm**	**Adults: Demographics Only**	**Adults: Demographics with Physical Intensity**	**Children: Demographics Only**	**Children: Demographics with Physical Intensity**
	**No. (%)**	**No. (%)**	**No. (%)**	**No. (%)**
Linear SVM	2404 (63.6)[a]	3310 (87.6)[a]	978 (50.4)[a]	1413 (72.8)[a]
Random Forest		3420 (90.5)[a]		1532 (78.9)[a]
**NHANES 2005-2006**				
**Machine Learning Algorithm**	**Adults: Demographics Only**	**Adults: Demographics with Physical Intensity**	**Children: Demographics Only**	**Children: Demographics with Physical Intensity**
	**No. (%)**	**No. (%)**	**No. (%)**	**No. (%)**
Linear SVM	2398 (62.8)[a]	3272 (85.8)[a]	977 (48.1)[a]	1439 (70.8)[a]
Random Forest		3428 (89.9)[a]		1538 (75.7)[a]

Abbreviations: NHANES, National Health and Nutrition Examination Survey; SVM = Support Vector Machine
[a] $P < 0.001$

© 2018 Na L et al. *JAMA Network Open.*
